# Anesthetic Management for Cesarean Delivery in a Patient with a Gigantic Intracranial Tumor

**DOI:** 10.1155/2020/9792580

**Published:** 2020-04-10

**Authors:** C. dos Santos e Santos, C. L. Mason, J. S. Neill, B. E. Grayson, A. Calimaran, D. R. Bacon

**Affiliations:** ^1^Department of Anesthesiology, University of Mississippi Medical Center, Jackson, MS, USA; ^2^Department of Pathology, University of Mississippi Medical Center, Jackson, MS, USA; ^3^Department of Neurobiology and Anatomical Sciences, University of Mississippi Medical Center, Jackson, MS, USA

## Abstract

A 31-year-old G5P1 patient with unremarkable past medical history at 29 weeks of gestation was diagnosed with a gigantic left frontotemporal brain mass. Initial clinical management as an inpatient achieved an improvement in the symptoms. The patient and surgical team agreed to schedule a cesarean delivery at 32 weeks of gestation if no neurological deterioration was observed. Intraoperative course with general endotracheal anesthesia and bilateral transversus abdominis plane block was uneventful and promoted efficient postoperative pain control. Seven days after delivery, the patient underwent craniotomy for brain tumor resection. This report describes the anesthetic management of a patient with an intracranial tumor during pregnancy.

## 1. Introduction

Central nervous system (CNS) tumors present in the general population at a low incidence and are generally classified as either (1) primary or metastatic and (2) nonmalignant or benign and malignant. The most prevalent primary CNS tumors in adults are meningiomas, pituitary adenomas, gliomas, Schwannomas, and pineal tumors in order of occurrence [1]. Even though CNS tumors are uncommon, they are responsible for high morbidity and mortality rates [2]. According to CBTRUS (Central Brain Tumor Registry of the United States), the incidence of CNS tumors in adolescents and young adults is 11.20 cases per 100,000 including both malignant and nonmalignant tumors [3]. Although females have a higher general incidence, malignant tumors are more prevalent in males than in females, corresponding to 55.4 and 44.6%, respectively [4]. In the U.S., gliomas are the most common histological type of malignant CNS tumors observed in females [5]. Even though CNS tumors during pregnancy are rarely observed, their biological behavior is unique in comparison to other CNS tumors in nonpregnant patients [6] and may be attributed to physiological changes related to hormonal alterations during pregnancy [7]. Despite the rarity of CNS tumors during pregnancy, when they do occur, they present multiple treatment dilemmas [8]. Though the patient ultimately makes the final decision to her treatment plan, the health professionals face the complicated work to disclose all the risks and benefits of her current clinical status and probable prognosis.

This case report obtained written permission from the patient to publish, and all authors either participated in the care of the patient or substantively helped to prepare this manuscript.

## 2. Case Description

**Figure 1:**
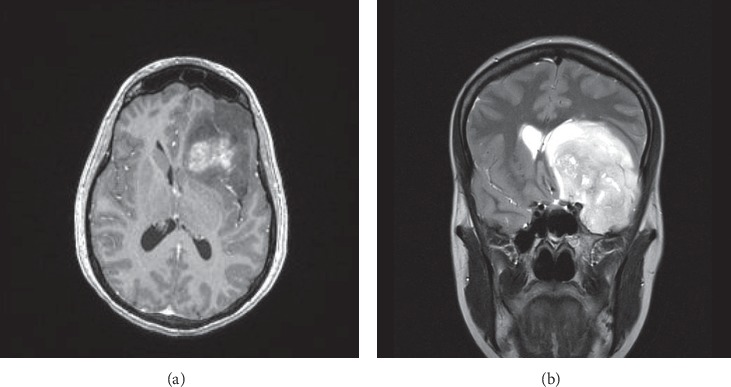
Brain magnetic resonance imaging, axial (a) and coronal (b) views, shows a large mass within the left anterior and middle cranial fossae with rightward midline shift on the underlying brain parenchyma (subfalcine herniation).

**Figure 2:**
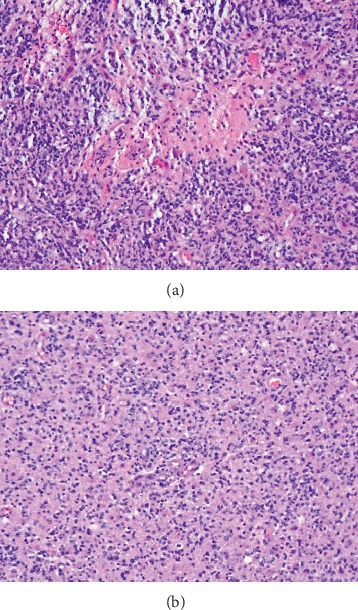
(a) Glioblastoma tumor shows high cellularity, cytological atypia, and necrosis. (b) Glial cell proliferation and vascular proliferation are present.

Here, we present successful anesthetic management of a patient with an intracranial tumor who underwent cesarean delivery (CD). We highlight the utility of transversus abdominis plane (TAP) blocks with liposomal bupivacaine in the provision of postcesarean analgesia when intrathecal analgesia is contraindicated. A 31-year-old gravida 5, para 1 (G5P1) with an unremarkable past medical history, at 29 weeks gestation was diagnosed with a left frontotemporal brain mass measuring 7 × 7 × 5.5 cm. Four months prior, the patient began therapy for migraine headaches that were occasionally accompanied by episodes of word-finding difficulty. After acute worsening and persistence of symptoms including nausea, appetite loss, and vision blurriness, the patient underwent magnetic resonance imaging (MRI) of the brain, which demonstrated the expansive intracranial process as well as subfalcine herniation (Figure 1) due to increased intracranial pressure (ICP). The patient was admitted to the Neurosurgery/Obstetrics services, and therapy was initiated with dexamethasone 4 mg every 6 hours until the cesarean section procedure to decrease cerebral perilesional edema and promote fetal lung maturity concomitantly. Levetiracetam and acetazolamide were added and maintained until after final home discharge to prevent seizures and decrease cerebral spinal fluid production, respectively. At that admission, the neurosurgeon discussed with the patient the potential for the mass to be malignant. The patient was informed that such a diagnosis would only be possible with either biopsy or surgical resection followed by pathologic analysis. After full disclosure by the Obstetrics and Neurosurgery teams, the patient decided to proceed with the pregnancy to 32 weeks of gestation. After eight days of in-house clinical management, she was discharged home, and a CD was scheduled at 32 weeks of gestation with possible anticipation if the neurological deterioration was observed. Without additional complications, the patient presented for elective CD. The presence of an intracranial mass leading to increased ICP contraindicated either neuraxial anesthesia or intrathecal analgesia. In the preoperative area, the patient and her family were informed about the anesthetic plan and risks of the procedure. Consent was obtained, and she was transferred to the operating room. A Standard American Society of Anesthesiologists monitor was placed, and preoxygenation initiated. All vital signs were stable and within normal limits. When the surgical team was ready to perform the CD, general endotracheal anesthesia was induced via rapid sequence to reduce the risk of bronchoaspiration [9]. The intravenous hypnotic agent used was propofol in a dose of 3 mg/kg. The neuromuscular blocker of choice was Rocuronium (1.2 mg/kg) since the patient already had increased ICP. The reason succinylcholine should be avoided in patients with elevated ICP has been extensively described in the literature [10]. After a healthy infant was born, 100 µg of fentanyl was administered. Endotracheal intubation was performed without complications, at the first attempt, using a MAC T3 blade attached to a video laryngoscope. The orogastric tube was placed, and the stomach contents aspirated. Bilateral ultrasom- (US-) guided TAP block with liposomal bupivacaine 1.3% was performed at the end of the surgical procedure. TAP block performed with 10 mL of liposomal bupivacaine 1.3% (133 mg) with additional 10 mL of normal saline injected in each side of the abdominal wall between the internal oblique and the transversus abdominis muscles without complications (total of 266 mg). Immediately after the bilateral peripheral nerve block, neuromuscular block reversal with glycopyrrulate and neostigmine was given, and the patient was successfully extubated in the operating room. Intra- and postoperative courses were uneventful. Bilateral TAP block was effective, and the patient required the first dose of morphine 28 hours after TAP block being performed. One week after CD, the patient underwent a successful craniotomy for the tumor resection. Pathology analysis demonstrated a glioblastoma WHO grade IV (Figure 2). Seven days later, the patient was discharged home neurologically intact. She is currently under Oncology treatment obtaining radiation therapy.

## 3. Discussion

Diagnosis of an intracranial tumor may be delayed in pregnancy, given that intracranial expansive-associated symptoms (headache, nausea, and vomiting) are frequent during pregnancy. Physiological changes of pregnancy, primarily related to increased levels of progesterone, may precipitate growth of intracranial tumors as well as trigger flaring of symptoms [11]. Assisted vaginal delivery may be achieved, but CD is performed if there is elevated intracranial pressure or an obstetrical indication [12]. Neurosurgical intervention during pregnancy will occur in cases where malignancy is suspected and where there is neurological deterioration due to either decompensated hydrocephalus requiring ventricular drainage or intracranial herniation. Bilateral TAP block may provide adequate postcesarean analgesia when intrathecal morphine is contraindicated. The addition of liposomal bupivacaine may increase the duration of the analgesia. US-guided TAP blocks following CD are reported as efficient if correctly executed [13], and the rules in facilitating extubation in patients with elevated intracranial pressure are well described in the literature [14].

This case demonstrates the effectiveness of US-guided TAP block following CD, mainly when neuraxial analgesia cannot be performed. It is indicated for postoperative pain control, increasing patient satisfaction, and reducing postoperative opioid consumption and its undesired side effects.
